# Direct Comparison of a Natural Loss-Of-Function Single Nucleotide Polymorphism with a Targeted Deletion in the *Ncf1* Gene Reveals Different Phenotypes

**DOI:** 10.1371/journal.pone.0141974

**Published:** 2015-11-03

**Authors:** Outi Sareila, Cecilia Hagert, Pia Rantakari, Matti Poutanen, Rikard Holmdahl

**Affiliations:** 1 Medicity Research Laboratory, University of Turku, Turku, Finland; 2 The National Doctoral Programme in Informational and Structural Biology, Turku, Finland; 3 Department of Physiology, Institute of Biomedicine, and Turku Center for Disease Modeling, University of Turku, Turku, Finland; 4 Medical Inflammation Research, Karolinska Institutet, Stockholm, Sweden; Queen Mary University of London, UNITED KINGDOM

## Abstract

The genetic targeting of mouse models has given insight into complex processes. However, phenotypes of genetically targeted mice are susceptible to artifacts due to gene manipulation, which may lead to misinterpretation of the observations. To directly address these issues, we have compared the immunological phenotypes of *Ncf1* knockout mice with *Ncf1* ^*m1J*^ mice possessing a naturally occurring intronic loss-of-function SNP in their *Ncf1* gene. Neutrophil cytosolic factor 1 (NCF1) is the key regulatory component of the phagocytic NADPH oxidase 2 (NOX2) complex. Defects in NCF1 lead to lower production of reactive oxygen species (ROS) associated with autoimmune diseases in humans. In mice, collagen induced arthritis (CIA) and psoriatic arthritis are autoimmune disorders known to be regulated by *Ncf1*, and they were utilized in the present study to compare the *Ncf1* knockout with *Ncf1* ^*m1J*^ mice. Targeted *Ncf1* knockout mice were generated on a pure C57BL/6N genetic background, and thereafter crossed with B10.Q.*Ncf1* ^*m1J*^ mice. The targeting silenced the *Ncf1* gene as intended, and both the B6N;B10.Q.*Ncf1* ^*m1J*^ mice as well as the knockout littermates had reduced ROS production compared to wild type mice. Both also exhibited enhanced STAT1 (signal transducer and activator of transcription 1) protein expression as an indicator of pronounced interferon signature reported recently for *Ncf1* deficient mice. Surprisingly, female *Ncf1* knockout mice were protected from CIA whereas the *Ncf1* ^*m1J*^ females developed severe disease. Ovariectomization retrieved the susceptibility of *Ncf1* knockout females pointing to a sex hormone regulated protection against CIA in these mice. The data partly explains the discrepancy of the phenotypes reported earlier utilizing the *Ncf1* ^*m1J*^ mice or *Ncf1* knockout mice. These observations indicate that even a targeted knockout mutation may lead to a different biological outcome in comparison to the natural loss-of-function mutation of the same gene.

## Introduction

The reported immunological phenotypes for the neutrophil cytosolic factor 1 (*Ncf1 alias p47phox*) knockout mice and *Ncf1* ^*m1J*^ mice differ leading to some controversy. Up to date, the commonly used *Ncf1* knockout mouse was originally generated using 129 ES cells, backcrossed to C57BL/6 mice, and used as a model of chronic granulomatous disease (CGD) [1]. These *Ncf1* knockout mice were also reported to be completely protected from experimental autoimmune encephalomyelitis (EAE), when the disease was induced by the myelin oligodendrocyte glycoprotein (MOG) peptide [2]. EAE is a murine model of multiple sclerosis, a chronic inflammatory autoimmune disease of the brain, and these results indicate that *Ncf1* does not contribute to the development of autoimmune inflammation in this model.

The naturally occurring *Ncf1* ^*m1J*^ loss-of-function mutation was first discovered in a C57BL/6J-m db/db mouse [3]. It was later backcrossed to a clean wild type background, such as C57BL/6 or B10.Q, and shown to remarkably increase susceptibility to the autoimmune symptoms of collagen induced arthritis (CIA) and EAE [4]. When EAE was induced with the native MOG protein, the BQ.*Ncf1* ^*m1J/m1J*^ mice developed more severe EAE than the wild types, in contrast to the EAE phenotype reported for *Ncf1* knockout mice [2]. Recently, *Ncf1* ^*m1J*^ mice were described to spontaneously develop a lupus-like phenotype on the Balb/c background [5]. These reports describing various models of chronic inflammatory autoimmune diseases emphasize the role of *Ncf1* in regulating the development of autoimmunity.

Gene knockouts are usually generated by large genetic modifications. In contrast, *Ncf1* ^*m1J*^ is a naturally occurring intronic mutation of a single nucleotide (A→C) at the –2 position of exon 8 of the *Ncf1* gene [3]. The intronic SNP leads to aberrant splicing of the *Ncf1* transcripts, resulting in three different transcript variants detected with RT-PCR and sequencing, and the expression of the aberrant NCF1 (alias P47^PHOX^) protein in trace amounts in bone marrow cells [3]. We have recently reported that the NCF1/P47^PHOX^ variant expressed in *Ncf1* ^*m1J*^ mice is defective in activating the NOX2 complex to produce ROS [6], and thus in terms of ROS production, it can be compared to a NOX2 knockout.

NCF1 (alias P47^PHOX^) is one of the activating components of the transmembrane NADPH oxidase 2 complex (NOX2; alias GP91^PHOX^), which produces superoxide into the extracellular or intraphagosomal space [7]. The NOX2 complex consists of transmembrane core components P22^PHOX^ and GP91^PHOX^ as the enzymatic core. NCF1, NCF2 and NCF4 (also called P47^PHOX^, P67^PHOX^ and P40^PHOX^, respectively) are the cytosolic regulatory components of the NOX2 complex together with the RAC GTPase [7]. Defects in any of the PHOX proteins may lead to CGD symptoms [8,9].

In addition to CGD, defects in NOX2 complex genes and the compromised capacity to produce ROS have been connected to the development of autoimmunity in humans. The *NCF1* gene copy number has been associated with human rheumatoid arthritis (RA) [10]. Genes coding for two other regulatory components of the NOX2 complex, namely P40^PHOX^ and P67^PHOX^, have also been associated with autoimmunity. A SNP (rs729749), located in intron 4 of the *NCF4* gene, coding for P40^PHOX^, was shown to be associated with RA in a subgroup of patients [11]. Polymorphism in the *NCF2* gene, coding for P67^PHOX^ protein was reported to be associated with systemic lupus erythematosus (SLE) and the determining single nonsynonymous coding mutation in exon 12 (rs17849502; H389Q) identified to cause the P67^PHOX^ defect [12,13]. In addition, SLE patients with a more severe organ damage had a lower oxidative burst by polymorphonuclear leukocytes [14]. Guillain–Barré syndrome is a demyelinating autoimmune disease, and patients with severe symptoms have lower oxygen radical production in peripheral blood leukocytes compared with that in patients with a milder disease [15]. These reports demonstrate the link between a lower oxidative burst and the development of autoimmunity in humans.

*Ncf1* ^*m1J*^ mice exhibit a defect in ROS production by the NOX2-complex, and they are used as models of autoimmunity. However, the reported immunological phenotypes of the *Ncf1* knockout mice and *Ncf1* ^*m1J*^ mice are controversial raising questions about how *Ncf1* and ROS act as immunomodulatory components. The present study describes for the first time a direct comparison of these two different *Ncf1*-defective alleles, with all other factors neutralized, in the development of autoimmunity and inflammation.

## Materials and Methods

### Generation of *Ncf1* knockout mice

C57BL/6J BAC clone, RP24-53E17, containing the mouse *Ncf1* gene was obtained from BACPAC Resources Center (Children´s hospital Oakland Research Institute, Oakland, (CHORI), California, USA). A 11 000 bp-long DNA fragment of the *Ncf1* gene containing exons one to six was subcloned to a minimal vector, pACYC177 (New England Biolabs, MA, USA) using the Red/ET cloning method (Gene Bridges GmbH, Germany). The PCR product used in subcloning was amplified from a pACYC177 plasmid using the primers (A) 5´-CTG GAT CTT GTT CTG TTC TGT AGA CCA GGC TGG CCT CCA GCT CAC AGA GAG GCA GAC CTC AGC GCT AG-3´ and (B) 5´-CCG GCC TCC ATC TCA GAC CTA TCC CGT GCC TAG ATT GCA TTA AGG GGC GCT GAA GAC GAA AGG GCC TC-3´ including homology arms for the *Ncf1* gene. Murine *Ncf1* cDNA was cloned by reverse transcription, amplified with PCR from Balb/c spleen total RNA and ligated to a pIRES2-EGFP plasmid (Clontech) resulting in the plasmid m*Ncf1*-IRES2-EGFP-NEO. The cassette FRT-lox66-m*Ncf1*-IRES2-EGFP-NEO-lox71-FRT containing 50 bp of *Ncf1* homology arms was used to destroy the mouse genomic *Ncf1* exons 1 and 2. The cassette was amplified by PCR and it was inserted in an inverted orientation using the Red/ET recombination method. Validity of the final targeting construct was confirmed by restriction enzyme digestion analysis and sequencing (S1 Fig).

JM8.N4 mouse embryonic stem cells were cultured on neomycin-resistant primary embryonic fibroblast feeder cells, and 10^6^ cells were electroporated with the linearized targeting construct. After electroporation, the cells were plated on culture dishes and exposed to G418 (Sigma). Colonies were picked after growing for 7–9 days in the selection media, and expanded by growing them on 96-well plates. The selected ES cell clones were screened by locus-specific PCR and southern blot, selectively identifying the ES cell clones with a properly inserted targeting vector. The targeted ES cells were injected into C57BL/6N mouse blastocysts, and chimeric mice were generated. By crossing the highly chimeric male mice with C57BL/6N females, a germline transmission was achieved, resulting in mice heterozygous for the deficiency of *Ncf1* (C57BL/6N-*Ncf1* ^*Tm2Utu*^, shortly B6N.TN2).

### Mice

Mice with the *Ncf1* knockout allele *Ncf1* ^*Tm2Utu*^ (B6N.TN2) were backcrossed for three generations to B10.Q.*Ncf1* ^*m1J*^ mice analyzed for the possession of only the causative mutation using a 10-k SNP typing chip. Those with a homozygous CIA susceptibility MHC II allele H2-Aq were selected for intercrossing in order to establish the B6N;B10.Q.TN2.*Ncf1* ^*m1J*^ line. Littermates carrying the homozygous *Ncf1* knockout allele (denoted KO) or the homozygous *Ncf1* ^*m1J*^ SNP (rs230824082; denoted *Ncf1* ^*m1J*^) were used in the experiments. In addition, mice of different genotypes were randomly housed, and treated and analyzed blindly. Treating and analyzing the mice was performed in a cage-wise order leading to clear randomization of the individuals in the experimental genotypic groups.

For experiments where three *Ncf1* alleles (*Ncf1* ^*m1J*^, KO and WT) were compared, mice with the KO allele were bred in two separate lines with *Ncf1* variants, *Ncf1* ^*m1J*^ and the WT allele, respectively, and the KO homozygotes were collected into the experiment from both lines. The wild type control group consisted of mice carrying either heterozygously (WT/KO) or homozygously (WT/WT) wild type *Ncf1*. Sex- and age-matched mice were used in the experiments. The mice were 7–14 weeks old and weighed 18–35 g at the start of the experiments, unless otherwise specified.

The mice were housed in wire-topped cages (2–6 mice per cage) with aspen chips bedding (Tapvei, Kaavi, Finland) under specific pathogen-free conditions in a climate-controlled environment with 12 h h/12 h light/dark cycles in the Central Animal Laboratory of University of Turku. Standard rodent chow (SDS, Special Diet Services, Whitham, Essex, UK) and tap water was provided *ad libitum*. Animal care was in accordance with the institutional guidelines with cage enrichment and the welfare of the animals assessed daily throughout the experiments. The national Animal Experiment Board in Finland (Eläinkoelautakunta, ELLA) approved this study: ethical permit numbers: ESAVI-0000497/041003/2011 and ESAVI/439/04.10.07/2017. Genotyping was done by PCR for the targeted *Ncf1* gene and by a SNP genotyping assay for the *Ncf1* ^*m1J*^ allele (Applied Biosystems, CA, USA) using genomic DNA from an earpiece.

Ovariectomies were performed by ligating and excising the ovaries through a single posterior-approach incision in female mice (mean age: 10 weeks; range: 8–11 weeks). Anesthesia and analgesia were induced preoperatively with an intraperitoneal injection of ketamine (Ketaminol vet, Intervet International B.V., Boxmeer, Netherlands; 75 mg/kg) and xylazin (Rompun vet, Bayer HealthCare AG, Leverkusen, Germany; 8 mg/kg) and during the operation with inhaled isoflurane. An adequate depth of anesthesia was verified by the absence of withdrawal reflexes. Buprenorphine (Temgesic, Schering-Plough Corp., Espoo, Finland; intraperitoneally, 0,05–1 mg/kg, twice a day for one day) was used as a postoperative analgesic. After the operation the mice recovered for two weeks before immunization. At the end of the experiments, animals were euthanized with carbon dioxide.

### Measurement of intracellular and extracellular ROS

For determination of the intracellular oxidative burst by dihydrorhodamine-123 fluorescence, the cells were exposed to phorbol myristate acetate (PMA; 200 ng/ml, 20 min) *ex vivo* according to the protocol described previously [16], and by applying the modifications recently described [17]. Data was obtained as previously described [6], and analyzed as an increase in the geometric mean of dihydrorhodamine-123 fluorescence in comparison to the solvent control. To pool data from independent experiments, ROS production in WT males in comparison to *Ncf1* ^*m1J*^ males (difference in Geometric mean) was set as a stimulation index for each experiment. The geometric mean of each sample was then normalized to the stimulation index within the same experiment, after which all data was pooled and presented as relative values.

The extracellular oxidative burst from cells in tissue homogenate was measured as previously reported [6]. During the preparation of single cell suspensions, red blood cells were lysed by isotonic lysis buffer, suspensions filtered through 70 μm strainers, and cell concentrations were equalized for the measurement.

### Flow cytometry

Flow cytometric analyses were performed as described previously [5]. Briefly, red blood cells were lysed by hypotonic buffer, Fc-receptors blocked and surface antigens stained with fluorescently labelled antibodies. For intracellular staining, the cells were fixed and permeabilized with Cytofix/Cytoperm™ solution (BD Biosciences) according to the manufacturer’s protocol. NCF1 expression was determined with mouse monoclonal anti-NCF1 antibody (clone D-10; Santa Cruz Biotechnology, Dallas, USA). Cells were acquired with LSR II (BD Biosciences) and the results analyzed with Flowing software (Cell Imaging Core, Turku Centre for Biotechnology, Finland). STAT1 expression was determined as an increase in mean fluorescence intensity in comparison to the isotype control as previously described [5]. When collecting the data from several independent experiments, the average of the STAT1 expression in *Ncf1* ^*m1J*^ cells was set as 100%, and all expression values determined were related to that within each experiment, after which the results were pooled.

### Collagen induced arthritis (CIA)

For CIA, both female (mean weight 22 g; range 17–28 g) and male (mean weight 29 g; range 19–35 g) mice (age between 10–13 weeks, unless otherwise stated) were used. Each mouse was immunized intradermally above the base of the tail with 100 μg of bovine CII (#804001; MD Biosciences, Zürich, Switzerland) emulsified in Complete Freund Adjuvant (CFA; Difco # 263810; BD Biosciences, New Jersey, US) in a total volume of 100 μl per mouse under isoflurane anesthesia. The mice were immunostimulated 21 days later similarly by an intradermal injection of 50 μg bovine type II collagen emulsified in Incomplete Freund adjuvant (IFA; #263910; BD Bioscience) in a volume of 50 μl per mouse. Clinical symptoms were followed by a standardized macroscopic scoring system by giving 1 point for each red and swollen toe/finger and metacarpo- and metatarsal phalangeal joint, in addition to 5 points for an inflamed ankle/wrist leading to a maximum of 15 points per paw and max 60 points per mouse. Immunizations and scoring was conducted blindly without knowing the genotype of the mice.

### Measurement of anti-collagen antibodies

Anti-collagen type II IgG antibody levels in the serum were analyzed by ELISA with small modifications to the previously described protocol [4]. Briefly, Nunc Maxisorp 96-well plates (Thermo Fischer Scientific Inc., Waltham, USA) were coated with rat pepsin-digested collagen type II (#8041006-B; MD Biosciences, Zürich, Switzerland). Plates were sequentially blocked with bovine serum albumin (fraction V; Immuno Diagnostic Oy, Hämeenlinna, Finland). All washings were performed with PBS containing 0.1% Tween20. Serum was diluted into PBS and incubated on bound collagen for 2 h at RT. Bound antibodies were detected with biotinylated Ig, κ light chain (clone 187.1; BD Biosciences) and streptavidin-conjugated Europium (PerkinElmer, Turku, Finland). Time resolved fluorescence was measured with Victor 1420 multi-label counter (PerkinElmer). Relative concentrations were determined by comparing the fluorescence signals of the samples to the standard curve obtained from pooled positive control serums. The concentration of anti-collagen type II antibodies in the pooled positive control serum was determined by comparing it to a dilution series of pooled four monoclonal anti-collagen type II (CII) antibodies with determined protein content [18]. When the data was pooled from independent experiments, all values were normalized to the mean value of *Ncf1* ^*m1J*^ (d21) serum within the same experiment, then pooled and presented as relative to *Ncf1* ^*m1J*^ values.

### Psoriatic inflammation

Mannan-induced psoriatic arthritis (PsA) was induced in mice (mean age: 13 weeks; range 2–4 months) as described recently. The paw inflammation was evaluated by a standardized macroscopic scoring system in a blinded manner [19]. Skin inflammation was analyzed in a blinded manner by visual scoring of the skin in the ears (0–3 points in total), and in the paws (0–3 points/paw) as follows: 1 = weak skin pealing, 2 = moderate skin pealing, 3 = heavy skin pealing. The scoring was combined to yield a maximum total score of 15.

### Statistical analyses

Disease scores were analyzed with the Mann-Whitney U-test, whereas cellular analyses were analyzed with one-way ANOVA combined with the Bonferroni posttest using GraphPad Prism Software (GraphPad Software, Inc., La Jolla, USA) unless otherwise stated. In all analyses, the number of independent observations (n) refers to the number of the analyzed mice or the number of mice the samples were collected from.

## Results and Discussion

### *Ncf1* knockout mice cannot produce ROS by the NOX2 complex

**Fig 1:**
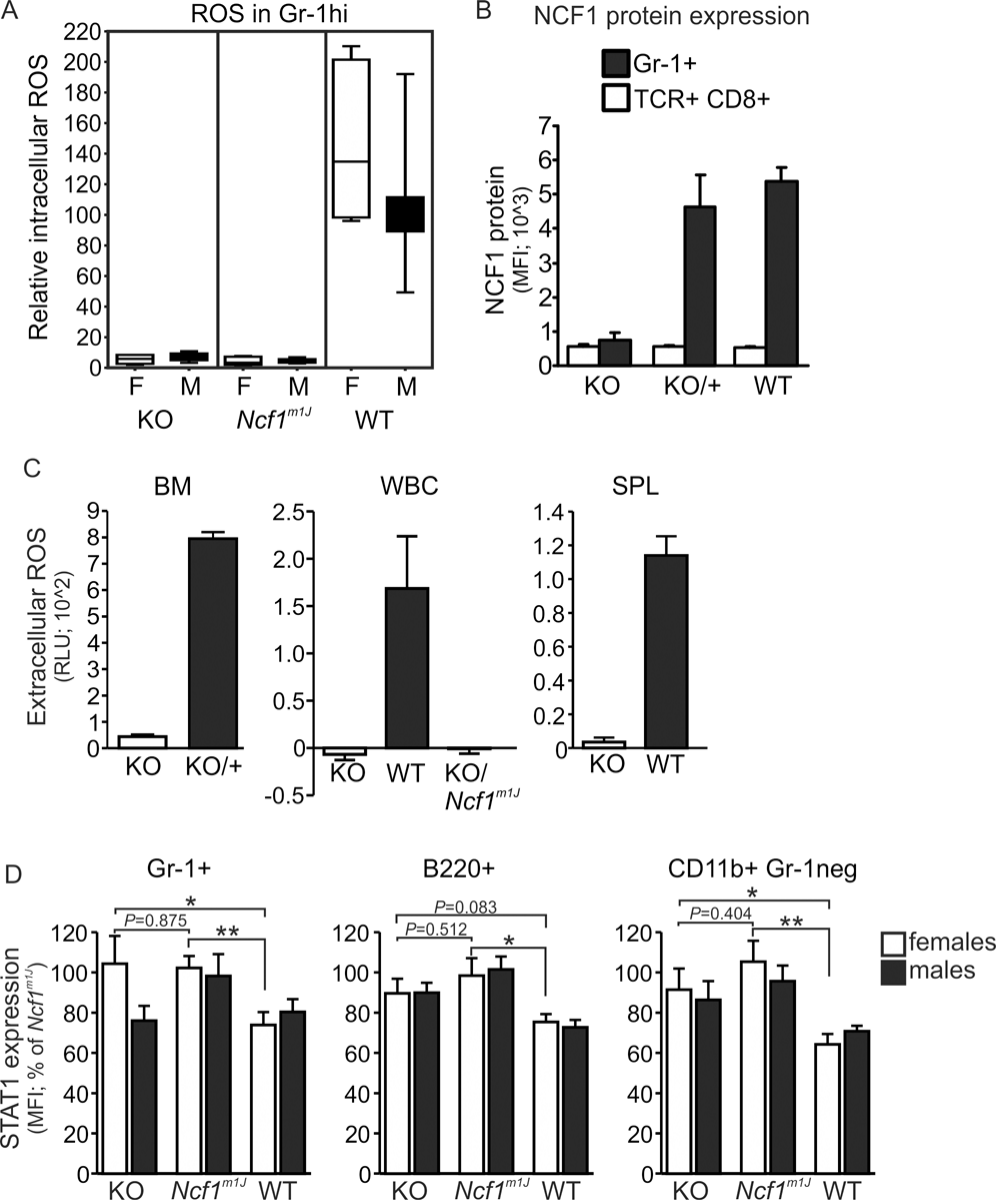
*Ncf1* knockouts and *Ncf1* ^*m1J*^ mice are deficient in ROS production and share the interferon signature. (A) Intracellular ROS production is absent in blood granulocytes from *Ncf1*-deficient mice. Blood leukocytes from *Ncf1* knockouts (KO), wild types (WT) and mice with the *Ncf1* ^*m1J*^ mutation were stained for surface markers and stimulated by PMA. Intracellular ROS production was quantified as dihydrorhodamine-123 fluorescence by flow cytometry. F = females, M = males. (B) *Ncf1* knockouts do not express the NCF1 protein in blood granulocytes (Gr-1+) when analyzed by intracellular staining and flow cytometry. T cells (CD3+CD8+) were used as negative control. (C) *Ncf1* knockouts fail to respond to PMA stimulation by extracellular ROS production. Bone marrow (BM), blood (WBC for white blood cells) and spleen (SPL) cell suspensions were depleted of erythrocytes and stimulated in the presence of isoluminol, a cell impermeable chemiluminescent substrate. Extracellular ROS production is presented as signal after 30 min stimulation. RLU = relative luminescence units. (D) STAT1 expression is upregulated in the absence of functional *Ncf1*. Blood cells from naïve mice were stained for cell surface markers. STAT1 expression was analyzed by intracellular staining, quantified by flow cytometry, and analyzed in granulocytic (Gr-1+) monocytic (CD11b+ Gr-1 negative) cells and B cells (B220+). The WT group comprises of mice with the wild type *Ncf1* either as homozygous or heterozygous (+/KO). Statistical significances between the female genotypes were calculated with unpaired Student’s t-test and presented as *P*-value or indicated as * *P*<0.05 and ** *P*<0.01. The data is presented as a box-and-whisker blot showing the quartiles (A) or as mean +/-SEM (B-D). Graphs (A), (SPL in C) and (D) present pooled data from three, two or four independent experiments, respectively. Number of mice per genotype: n = 6–12 in A; n = 4–5 in B; n = 4–15 in C; n = 6–13 in D. MFI = mean fluorescence intensity.

The NOX2 complex activation and subsequent ROS production is *Ncf1*dependent [20]. We compared the ROS production capability of cells extracted from the newly generated *Ncf1* knockout (KO) with the cells from *Ncf1* ^*m1J*^ mutant mice. No differences in the intracellular ROS production could be found between these strains, or between males and females (Fig 1A). Similar results were obtained from the immunized mice by analyzing the blood cells collected at the time of the termination of the CIA experiment (S2 Fig). Successful inactivation of the *Ncf1* gene in the KO mice was verified on protein level with intracellular staining of blood granulocytes (Fig 1B), known to express high levels of the NCF1 protein [20], as well as by measuring extracellular ROS production (Fig 1C).

### Interferon signature in both *Ncf1* knockouts and *Ncf1* ^*m1J*^ mice

*Ncf1* ^*m1J*^ mice display an upregulation of interferon regulated genes, and one of the characteristic over-expressed proteins is STAT1 [5]. We investigated whether ROS deficiency leads to the same phenotype in *Ncf1* KO mice. Similar to the *Ncf1* ^*m1J*^ mice, upregulated STAT1 expression was found in *Ncf1* KO female mice (Fig 1D). Likewise, STAT1 upregulation was observed in B220+ and CD11b+Gr-1neg cells in male *Ncf1* KO mice. In Gr-1+ cells from the *Ncf1* KO males, the expression did not seem to differ from the wild type, although it did not reach statistical significance when compared with Gr-1+ cells from the *Ncf1* KO females. These results point to a similar cellular phenotype in these mice caused by ROS-deficiency in females in all analyzed blood cell types.

### *Ncf1* knockout females are protected from CIA

**Fig 2:**
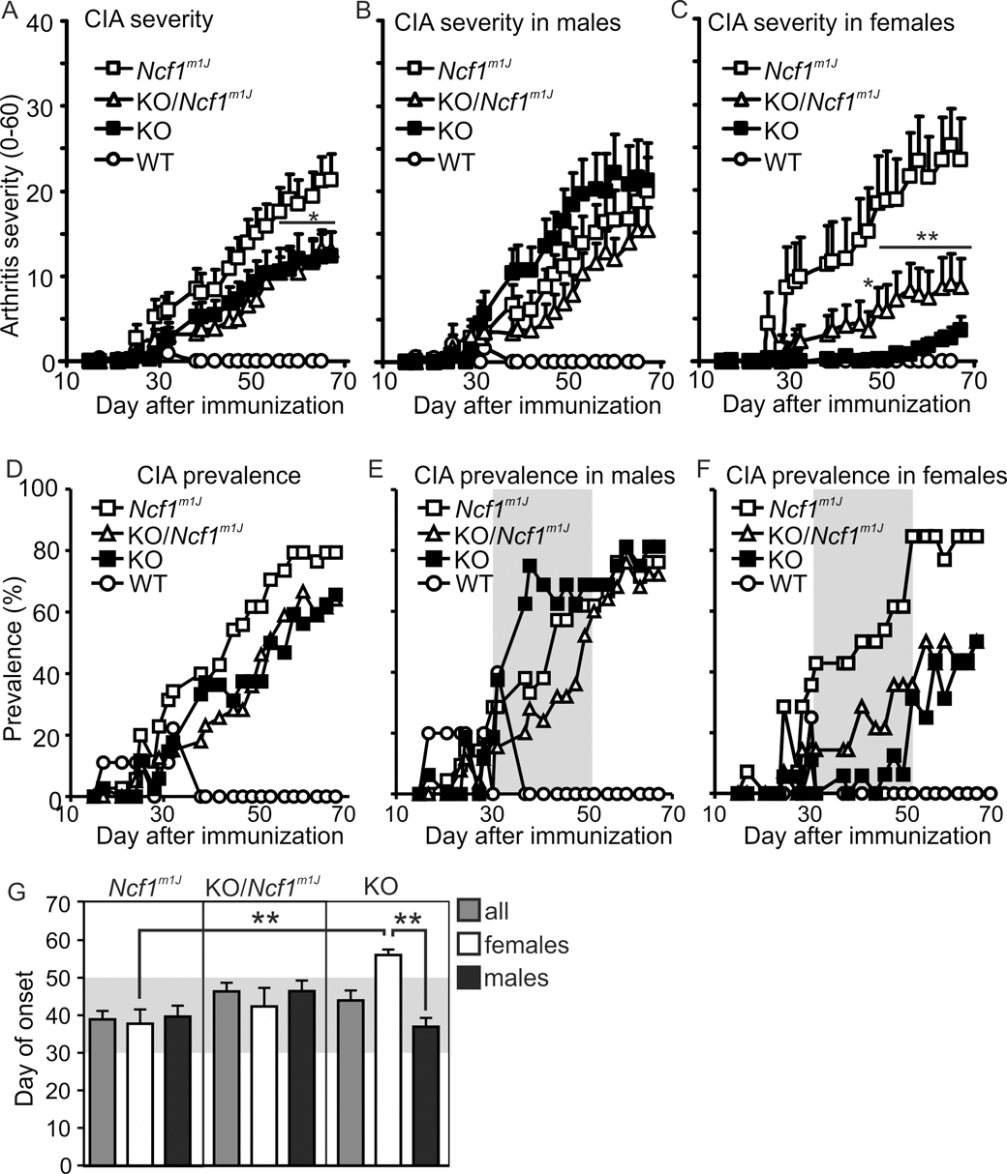
Comparison of collagen induced arthritis (CIA) in *Ncf1* knockout and *Ncf1* ^*m1J*^ mice. Arthritis severity was evaluated by macroscopic scoring of paw inflammation (see methods for details) and presented by genotype (A) or shown separately for males (B) and females (C). Prevalence of paw inflammation is presented by genotype (D) or shown separately for males (E) and females (F). The data is pooled from two identically performed experiments with similar results. The wild type littermate controls were included only in the first experiment. Statistical significances between the knockout (KO) and *Ncf1* ^*m1J*^ mice were calculated with Mann-Whitney U-test and presented as ** *P*<0.01, * *P*<0.05. (G) The mean day of disease onset after immunization is presented by genotype and gender. Significant difference in the day of onset in comparison to the group of *Ncf1* KO females was calculated by One-way ANOVA with Dunnett’s posttest. ** *P*<0.01. The data is presented as mean (+/-SEM in A–C and G). Number of mice per genotype: n = 32–39 in A and D, n = 16–25 in B and E, n = 13–16 in C and F, n = 6–27 in G (except n = 4–9 for the WT). The grey box in E–G highlights the disease phase between days 30 and 50 post immunization.

Several NOX2 complex genes, including *NCF1*, have been found to be associated with autoimmune manifestations in humans [10,11,13]. Therefore, *Ncf1* deficient mice are utilized as models of autoimmunity. However, the reported immunological phenotypes for *Ncf1* KO and *Ncf1* ^*m1J*^ mice have been controversial. CIA is the most widely used model for rheumatoid arthritis, and it is the best characterized model describing the association of the *Ncf1* ^*m1J*^ and a lower oxidative burst with more severe arthritis [4]. Therefore, we compared *Ncf1* ^*m1J*^ mice to *Ncf1* KO littermates in CIA severity and susceptibility (Fig 2).

The *Ncf1* KO mice were susceptible to CIA, however, they developed a milder disease than their *Ncf1* ^*m1J*^ littermates (Fig 2A). When analyzing the influence of gender, the *Ncf1* KO males developed CIA with similar severity and prevalence as their *Ncf1* ^*m1J*^ littermates (Fig 2B and 2E). In contrast, the *Ncf1* KO females were highly protected from arthritis with a significantly milder disease (Fig 2C), lower prevalence (44% vs. 85%; Fig 2F and S1 Table) and remarkably later onset of the disease (Fig 2G and S1 Table) than their *Ncf1* ^*m1J*^ female littermates. The difference could not be explained by dissimilarities in cellular interferon signature (Fig 1D) or in ROS production in naïve mice nor during the CIA (Fig 1A and S2 Fig), although these traits have been reported to explain the increased severity and susceptibility to autoimmune diseases in mice and rats possessing mutations in the *Ncf1* gene [4,5,17,21].

**Fig 3:**
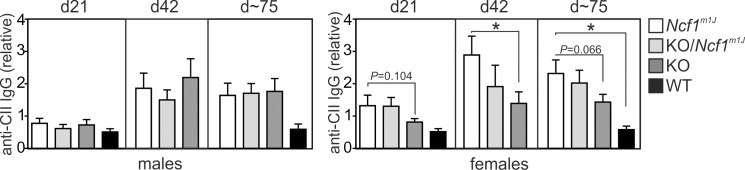
Antibody (IgG) response to type II collagen (CII) in the serum during CIA. Number of days post immunization is indicated above (d~75 referring to sera collected either 71 or 77 days after the immunization). The data (d21 and d~75) were pooled from two independent experiments. Day 42 (d42) data was only available from one of those experiments. * *P*<0.05 when calculated by Student’s t-test. The results are presented as mean +/-SEM, n = 4–25.

The humoral immune response during CIA was evaluated by determining the quantity of anti-collagen type II (CII) antibodies in the serum. *Ncf1* ^*m1J*^ female mice had more anti-CII antibodies than the *Ncf1* KO females during the disease (day 42 post immunization), and the tendency was observed at every time point analyzed (Fig 3, right panel), thus, reflecting the differences in the severity of the disease. In contrast, the nature of the *Ncf1* deficiency did not alter the anti-CII antibody levels in males (Fig 3, left panel).

CIA is a well-characterized, widely used model of rheumatoid arthritis known to be dependent on both T- and B-cells, macrophage responses [22], and regulated by the *Ncf1* gene [4]. To investigate whether the *Ncf1* KO female mice are, in general, resistant to the development of arthritic symptoms, the *Ncf1* KO and *Ncf1* ^*m1J*^ mice were exposed to mannan-induced psoriatic arthritis (PsA). The *Ncf1* ^*m1J*^ mutation exacerbates PsA in which neutrophil infiltration and interleukin-17 produced by γδ-T cells regulate the arthritic symptoms independently of the adaptive immune cells (αβT- and B-cells) [19]. Hence, the inflammatory mechanisms involved differ from those in CIA. Both *Ncf1* KO and *Ncf1* ^*m1J*^ mice developed severe paw inflammation and psoriatic lesions with similar severity and prevalence in both genders irrespective of the type of *Ncf1* deficiency (S3 Fig). These results indicate that the *Ncf1* KO females are specifically protected from collagen type II induced autoimmune arthritis, but are susceptible to another type of arthritis and develop symptoms as severe as their *Ncf1* ^*m1J*^ littermates.

### Possible factors influencing the phenotypic divergence between *Ncf1* knockout and *Ncf1* ^*m1J*^ mice

We studied littermate controls in the experiments to minimize differences between the different strains. The *Ncf1* KO was made in ES cells derived from the C57Bl/6N mouse strain, and the *Ncf1* ^*m1J*^ mutation occurred for the first time in a C57Bl/6 background. Both of them have been backcrossed to B10.Q, however, there could still be variable generic polymorphisms in the genome. Nonetheless, the littermate experimental setting should have excluded possible bias. The physical environment was well controlled, and further, we minimized the influence of environmental factors by housing the different genotypes mixed in cages. We believe that biased genetic or environmental factors explain most of the artifacts reported for knockout experiments conducted with animals derived from ES cells originating from the 129 strain, including the previous *Ncf1* knockouts [23]. Thus, we emphasized on excluding such simple explanations while still finding clear phenotypic differences. We believe the remaining factors that could possibly explain the difference are: (1) possible polymorphism in the chromosomal fragment linked to the *Ncf1* locus, (2) disturbance of the regulatory *cis-* or *trans-*elements within the knockout locus caused by a deletion of genomic DNA and/or an insertion of foreign DNA, (3) the untranslated RNAs of *Ncf1* ^*m1J*^ and *Ncf1* KO differ and may have variant functional effects, and finally (4) the defective but still existing NCF1 coded by the *Ncf1* ^*m1J*^ gene, which could give feedback regulation or still interact with other proteins than those regulating ROS production by the NOX2 complex.

To knock out the *Ncf1* gene, we generated a disruption cassette into the C57BL/6J BAC clone and targeted JM8.N4 (C57BL/6N) ES cells by replacing exons 1–2 with the cassette in a reverse orientation. When comparing the reported phenotypes of different *Ncf1* mutants or knockouts, the possible influence of linked SNPs in the surrounding genes cannot be excluded, since the 129, C57BL/6 and CBA genetic backgrounds differ remarkably [24]. To minimize this factor, we backcrossed the B6N *Ncf1* knockout mice for three generations to B10.Q.*Ncf1* ^*m1J*^ mice, which had been analyzed for the possession of only the causative *Ncf1* ^*m1J*^ mutation using a 10-k SNP typing chip, and thereafter used *Ncf1* ^*m1J*^ and *Ncf1* KO littermates in the experiments.

Seven transcript variants of the wild type *Ncf1* gene have been reported (ENSMUSG00000015950; Ensembl release 82; [25]), of which three are protein coding splice variants. All of the protein coding variants, in addition to one transcript considered to be subjected to non-sense-mediated decay, include exons 1 and 2. In order to generate the knockout, we deleted exons 1 and 2 in the *Ncf1* gene to prohibit expression of all of these splice variants (S1 Fig), and to prohibit the expression of another transcript variant generated by intron retention and coded by exon 2. We cannot exclude the possibility that another of two alternatively spliced *Ncf1* transcript variants, not believed to be expressed as protein, could still exist in the knockout generated for this study. On the other hand, the *Ncf1* ^*m1J*^ mutation, results in aberrant splicing and three alternatively spliced *Ncf1* mRNA variants have been reported to be expressed [3]. The difference in the repertoire of *Ncf1* RNAs expressed in *Ncf1* KO and *Ncf1* ^*m1J*^ mice could in theory explain the sex specific difference observed in the susceptibility of female mice to CIA, even though the mice were equally defective in producing intra- or extracellular ROS. Nevertheless, to our knowledge, non-sense mediated decay of RNA has not been reported to influence the development of CIA.

The mouse *Ncf1* gene is located on chromosome 5 (reverse strand) and partially overlaps the *Gtf2ird2* (General transcription factor II-I repeat domain-containing protein 2) gene on the forward strand. The *Ncf1* ^*m1J*^ SNP resides within the *Gtf2ird2* transcript variant, which is thought to undergo non-sense mediated decay (ENSMUST00000123941; Ensembl release 82; [25]). In Williams-Beuren syndrome, a rare neurodevelopmental disorder, *GTF2IRD2* is deleted, yet these patients do not seem to have an increased risk for developing autoantibodies [26]. Our previous reports describing enhanced susceptibility to autoimmunity in both mice and rats deficient of *Ncf1* [4,21] as well as the genetic association of *NCF1* and *NCF4* in RA in humans [10,11] demonstrate that ROS deficiency predisposes autoimmunity. In this study, CIA susceptibility was found to be similar in males and females with the *Ncf1* ^*m1J*^ SNP, and the *Ncf1* knockout females were the only ROS deficient mice protected from CIA. Thus, it is very unlikely that alterations in the non-sense mediated decay of the *Gtf2ird2* transcript could explain the observed sex specific difference in the susceptibility to CIA.

It is well known that both age and sex regulate the severity of symptoms in CIA in mice as a higher age and male sex lead to increased disease severity. [27,28]. We do not know the specific reason for *Ncf1* KO and *Ncf1* ^*m1J*^ females being more dissimilar in CIA susceptibility than the corresponding males in the present study. To our knowledge similar sex-specific differences between *Ncf1* deficient mice have not been reported. Often the sexes of the experimental animals are not even stated making it challenging to compare our results to published data. Thus our study is unique in comparing directly the *Ncf1* ^*m1J*^ and *Ncf1* KO littermates and both sexes in a model of autoimmune disease.

### Ovarian hormones regulate the protection in *Ncf1* knockout females in CIA

Female sex hormones have been found to suppress CIA in mice [29], and estradiol treatment reduces both the severity and prevalence of CIA in DBA/1 mice [30]. The effect of estrogens is mediated by the nuclear estrogen receptors in B10.Q x DBA/1 F1 mice [31]. In B10.Q.*Ncf1* ^*m1J*^ mice, in which CIA is a chronic relapsing disease, raloxifene (a selective estrogen receptor modulator) and estradiol treatments, as well as endogenous estrogens, decrease arthritis prevalence, prevent joint destruction and even counter generalized osteoporosis [32].

**Fig 4:**
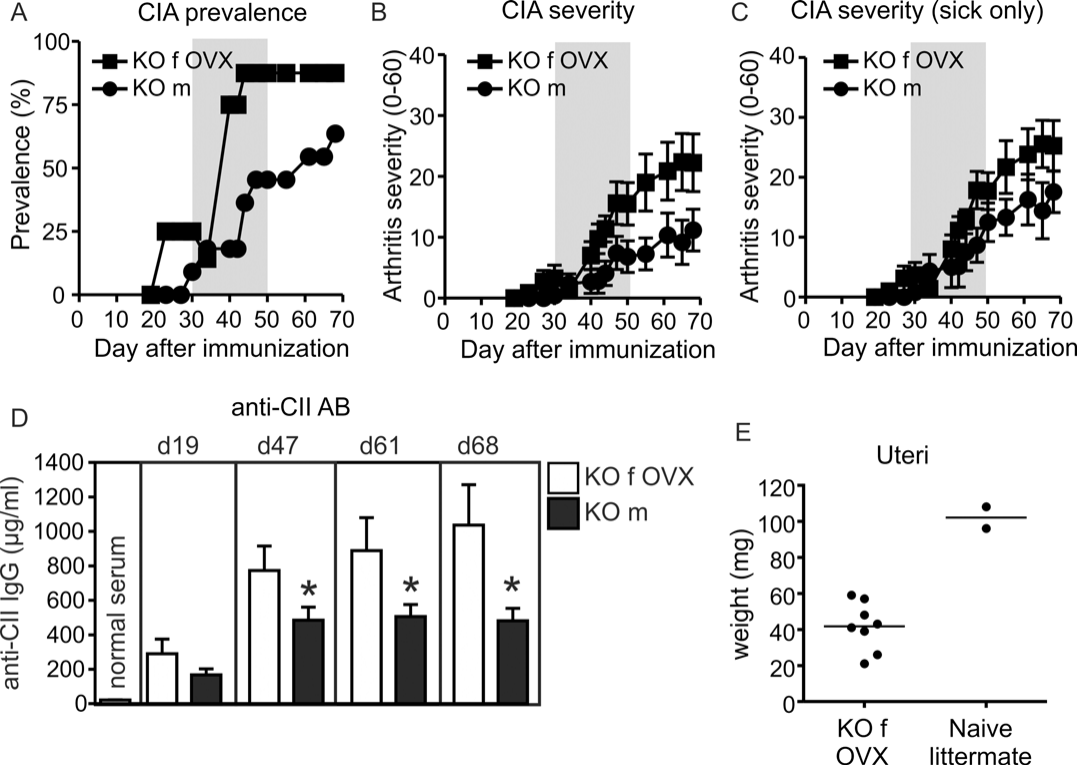
Ovariectomized *Ncf1* knockout females developed severe CIA similar to the knockout male littermates. The prevalence of paw inflammation is presented in A, and the severity of the disease in B. The severity of the disease after exclusion of the animals that never developed any symptoms is shown in C. Anti-collagen antibodies in the serum during CIA reflects disease severity (D). The uteri were weighed after euthanasia to verify successful removal of the ovaries (E). m = males, f = females, OVX = ovariectomized. Statistical significances between the genders in (D) were calculated by unpaired Student’s t-test (*P<0.05). The grey box (in A–C) highlights the disease phase between days 30 and 50 post immunization. The data is presented as mean +/-SEM in B-D and n = 8–11 in A-B and D, n = 7 in C.

To investigate whether the protected phenotype of the *Ncf1* KO females in CIA was due to ovarian hormones, we ovariectomized the females before they were subjected to CIA. Successful removal of the ovaries was assessed by weighing the uteri at the end of the experiment (Fig 4E). Almost all of the ovariectomized females developed CIA 30–50 days after immunization (Fig 4A), and the severity of CIA in females was similar to that of male littermates (Fig 4B–4C). Furthermore, the antibody response against type II collagen in the serum reflected the severity of inflammatory symptoms in the joints (Fig 4D). These results show that the protective effect was mediated via the ovarian hormones, since removal of the ovaries broke the tolerance against CIA in *Ncf1* KO females.

## Conclusions

The observed difference in the CIA phenotype between the two *Ncf1-*deficient genotypes highlights the important fact that genetic manipulation may influence complex phenotypes in a way that differs from that observed in a natural mutant. Obviously there are even more common reasons for different outcomes related to differences in genetic backgrounds (such as comparing mice with different genomic backgrounds instead of using true littermates) or differences in environmental setting (such as keeping mice of different genotypes in different cages while running experiments). Evidently, only sex- and age-matched littermates or fully backcrossed animals, which are preferably housed mixed in cages and in a well-controlled environment [33] should be used. Other factors like the gender of the animal technician could influence the outcome of an experiment, and even enrichment in the cages influences the variation in mouse models of arthritis [34] and osteoarthritis [35].

However, even when all such precautions are taken into consideration, our data shows that difference due to detailed genetic changes, introduced by technical manipulations, could influence a complex phenotype. Even though utilization of the transgenic technology has provided a huge advantage to study genes *in vivo*, this technique is vulnerable to artifacts. As found in the present study, even different loss-of-function modifications of the same genetic locus can lead to contrasting phenotypes. Even more recently, introduced technologies like CrispR (clustered regularly interspaced short palindromic repeats) could raise concerns, such as the introduction of off-target mutations [36]. The *Ncf1* ^*m1J*^ mouse, used in the present study, has demonstrated that a single naturally occurring point mutation in the intron [3] may lead to dramatic changes in the disease phenotype [4,5,37]. Such mutations may spontaneously occur in any strain at any time, which is another argument why littermate controls should always be used in scientific experiments.

## Supporting Information

S1 ARRIVE Checklist NC3Rs ARRIVE Guidelines Checklist Sareila.pdf. (PDF)

S1 Fig The targeting strategy of the *Ncf1* (neutrophil cytosolic factor 1) gene. (PDF)

S2 Fig ROS production by *Ncf1* deficient (KO and *Ncf1* ^*m1J*^) and the wild type mice during CIA. (PDF)

S3 Fig *Ncf1* deficient (KO and *Ncf1* ^*m1J*^) mice develop similar disease when exposed to psoriatic arthritis (PsA). (PDF)

S1 Table CIA in B6N;B10.Q mice with *Ncf1* deficiency. (PDF)

## Abbreviations

CGD: chronic granulomatous disease
CIA: collagen induced arthritis
CII: type II collagen
EAE: experimental autoimmune encephalomyelitis
KO: knockout
MFI: mean fluorescence intensity
MOG: myelin oligodendrocyte glycoprotein
NCF: neutrophil cytosolic factor
NOX2: NADPH oxidase 2
PsA: psoriatic arthritis
ROS: reactive oxygen species
SLE: systemic lupus erythematosus
SNP: single nucleotide polymorphism
STAT1: signal transducer and activator of transcription 1

## References

[1] Jackson SH, Gallin JI, Holland SM. The p47phox mouse knock-out model of chronic granulomatous disease. J Exp Med 1995 9 1;182(3):751–758. 7650482 10.1084/jem.182.3.751 PMC2192153

[2] van der Veen RC, Dietlin TA, Hofman FM, Pen L, Segal BH, Holland SM. Superoxide prevents nitric oxide-mediated suppression of helper T lymphocytes: decreased autoimmune encephalomyelitis in nicotinamide adenine dinucleotide phosphate oxidase knockout mice. J Immunol 2000 5 15;164(10):5177–5183. 10799876 10.4049/jimmunol.164.10.5177

[3] Huang CK, Zhan L, Hannigan MO, Ai Y, Leto TL. P47(phox)-deficient NADPH oxidase defect in neutrophils of diabetic mouse strains, C57BL/6J-m db/db and db/+. J Leukoc Biol 2000 2;67(2):210–215. 10670582 10.1002/jlb.67.2.210

[4] Hultqvist M, Olofsson P, Holmberg J, Bäckström BT, Tordsson J, Holmdahl R. Enhanced autoimmunity, arthritis, and encephalomyelitis in mice with a reduced oxidative burst due to a mutation in the Ncf1 gene. Proceedings of the National Academy of Sciences of the United States of America 2004 8 24;101(34):12646–12651. 15310853 10.1073/pnas.0403831101 PMC515111

[5] Kelkka T, Kienhofer D, Hoffmann M, Linja M, Wing K, Sareila O, et al Reactive Oxygen Species Deficiency Induces Autoimmunity with Type 1 Interferon Signature. Antioxid Redox Signal 2014 7 29. 10.1089/ars.2013.5828 PMC4224049 24787605

[6] Sareila O, Jaakkola N, Olofsson P, Kelkka T, Holmdahl R. Identification of a region in p47phox/NCF1 crucial for phagocytic NADPH oxidase (NOX2) activation. J Leukoc Biol 2013 3;93(3):427–435. 10.1189/jlb.1211588 23271700 PMC3579024

[7] Lambeth JD. NOX enzymes and the biology of reactive oxygen. Nat Rev Immunol 2004 3;4(3):181–189. 15039755 10.1038/nri1312

[8] Dinauer MC, Orkin SH. Chronic Granulomatous Disease. Annu Rev Med 1992 2/01;43(1):117–124. 1316094 10.1146/annurev.me.43.020192.001001

[9] Matute JD, Arias AA, Wright NAM, Wrobel I, Waterhouse CCM, Li XJ, et al A new genetic subgroup of chronic granulomatous disease with autosomal recessive mutations in p40phox and selective defects in neutrophil NADPH oxidase activity. Blood 2009 10 8;114(15):3309–3315. 10.1182/blood-2009-07-231498 19692703 PMC2759653

[10] Olsson LM, Nerstedt A, Lindqvist AK, Johansson AC, Medstrand P, Olofsson P, et al Copy Number Variation of the Gene NCF1 Is Associated with Rheumatoid Arthritis. Antioxid Redox Signal 2011 8 23. 10.1089/ars.2011.4013 21728841

[11] Olsson L, Lindqvist A, Kallberg H, Padyukov L, Burkhardt H, Alfredsson L, et al A case-control study of rheumatoid arthritis identifies an associated single nucleotide polymorphism in the NCF4 gene, supporting a role for the NADPH-oxidase complex in autoimmunity. Arthritis Research & Therapy 2007;9(5):R98. 17897462 10.1186/ar2299 PMC2212587

[12] Gateva V, Sandling JK, Hom G, Taylor KE, Chung SA, Sun X, et al A large-scale replication study identifies TNIP1, PRDM1, JAZF1, UHRF1BP1 and IL10 as risk loci for systemic lupus erythematosus. Nat Genet 2009 11;41(11):1228–1233. 10.1038/ng.468 19838195 PMC2925843

[13] Jacob CO, Eisenstein M, Dinauer MC, Ming W, Liu Q, John S, et al Lupus-associated causal mutation in neutrophil cytosolic factor 2 (NCF2) brings unique insights to the structure and function of NADPH oxidase. Proc Natl Acad Sci U S A 2012 1 10;109(2):E59–67. 10.1073/pnas.1113251108 22203994 PMC3258621

[14] Bengtsson AA, Pettersson A, Wichert S, Gullstrand B, Hansson M, Hellmark T, et al Low production of reactive oxygen species in granulocytes is associated with organ damage in systemic lupus erythematosus. Arthritis Res Ther 2014 6 5;16(3):R120 10.1186/ar4575 24902963 PMC4075132

[15] Mossberg N, Andersen O, Nilsson S, Dahlgren C, Hellstrand K, Lindh M, et al Oxygen radical production and severity of the Guillain–Barré syndrome. J Neuroimmunol 2007 12;192(1–2):186–191. 17945354 10.1016/j.jneuroim.2007.09.020

[16] Vowells SJ, Sekhsaria S, Malech HL, Shalit M, Fleisher TA. Flow cytometric analysis of the granulocyte respiratory burst: a comparison study of fluorescent probes. J Immunol Methods 1995 1 13;178(1):89–97. 7829869 10.1016/0022-1759(94)00247-t

[17] Hultqvist M, Sareila O, Vilhardt F, Norin U, Olsson LM, Olofsson P, et al Positioning of a polymorphic quantitative trait nucleotide in the Ncf1 gene controlling oxidative burst response and arthritis severity in rats. Antioxid Redox Signal 2011 6 15;14(12):2373–2383. 10.1089/ars.2010.3440 21275845

[18] Nandakumar KS, Holmdahl R. Efficient promotion of collagen antibody induced arthritis (CAIA) using four monoclonal antibodies specific for the major epitopes recognized in both collagen induced arthritis and rheumatoid arthritis. J Immunol Methods 2005 9;304(1–2):126–136. 16125192 10.1016/j.jim.2005.06.017

[19] Khmaladze I, Kelkka T, Guerard S, Wing K, Pizzolla A, Saxena A, et al Mannan induces ROS-regulated, IL-17A-dependent psoriasis arthritis-like disease in mice. Proc Natl Acad Sci U S A 2014 9 2;111(35):E3669–78. 10.1073/pnas.1405798111 25136095 PMC4156700

[20] El-Benna J, Dang PM, Gougerot-Pocidalo MA, Marie JC, Braut-Boucher F. p47phox, the phagocyte NADPH oxidase/NOX2 organizer: structure, phosphorylation and implication in diseases. Exp Mol Med 2009 4 30;41(4):217–225. 10.3858/emm.2009.41.4.058 19372727 PMC2679237

[21] Olofsson P, Holmberg J, Tordsson J, Lu S, Åkerström B, Holmdahl R. Positional identification of Ncf1 as a gene that regulates arthritis severity in rats. Nat Genet 2003 01;33(1):25 12461526 10.1038/ng1058

[22] Brand DD, Latham KA, Rosloniec EF. Collagen-induced arthritis. Nat Protoc 2007;2(5):1269–1275. 17546023 10.1038/nprot.2007.173

[23] Vanden Berghe T, Hulpiau P, Martens L, Vandenbroucke RE, Van Wonterghem E, Perry SW, et al Passenger Mutations Confound Interpretation of All Genetically Modified Congenic Mice. Immunity 2015 7 21;43(1):200–209. 10.1016/j.immuni.2015.06.011 26163370 PMC4800811

[24] Petkov PM, Ding Y, Cassell MA, Zhang W, Wagner G, Sargent EE, et al An efficient SNP system for mouse genome scanning and elucidating strain relationships. Genome Res 2004 9;14(9):1806–1811. 15342563 10.1101/gr.2825804 PMC515327

[25] Cunningham F, Amode MR, Barrell D, Beal K, Billis K, Brent S, et al Ensembl 2015. Nucleic Acids Res 2015 1;43(Database issue):D662–9. 10.1093/nar/gku1010 25352552 PMC4383879

[26] Stagi S, Lapi E, D'Avanzo MG, Perferi G, Romano S, Giglio S, et al Coeliac disease and risk for other autoimmune diseases in patients with Williams-Beuren syndrome. BMC Med Genet 2014 5 23;15:61-2350-15-61. 10.1186/1471-2350-15-61 PMC4035725 24885139

[27] Wilson-Gerwing TD, Pratt IV, Cooper DM, Silver TI, Rosenberg AM. Age-related differences in collagen-induced arthritis: clinical and imaging correlations. Comp Med 2013;63(6):498–502. 24326225 PMC3866989

[28] Holmdahl R, Jansson L, Gullberg D, Rubin K, Forsberg PO, Klareskog L. Incidence of arthritis and autoreactivity of anti-collagen antibodies after immunization of DBA/1 mice with heterologous and autologous collagen II. Clin Exp Immunol 1985 12;62(3):639–646. 4085150 PMC1577464

[29] Holmdahl R, Jansson L, Andersson M. Female sex hormones suppress development of collagen-induced arthritis in mice. Arthritis Rheum 1986 12;29(12):1501–1509. 3801072 10.1002/art.1780291212

[30] Holmdahl R, Jansson L, Meyerson B, Klareskog L. Oestrogen induced suppression of collagen arthritis: I. Long term oestradiol treatment of DBA/1 mice reduces severity and incidence of arthritis and decreases the anti type II collagen immune response. Clin Exp Immunol 1987 11;70(2):372–378. 3501349 PMC1542083

[31] Jansson L, Holmdahl R. Enhancement of collagen-induced arthritis in female mice by estrogen receptor blockage. Arthritis Rheum 2001 9;44(9):2168–2175. 11592382 10.1002/1529-0131(200109)44:9<2168::aid-art370>3.0.co;2-2

[32] Jochems C, Islander U, Erlandsson M, Engdahl C, Lagerquist M, Gjertsson I, et al Role of endogenous and exogenous female sex hormones in arthritis and osteoporosis development in B10.Q-ncf1*/* mice with collagen-induced chronic arthritis. BMC Musculoskelet Disord 2010 12 16;11:284-2474-11-284. 10.1186/1471-2474-11-284 PMC3009959 21159208

[33] Holmdahl R, Malissen B. The need for littermate controls. Eur J Immunol 2012 1;42(1):45–47. 10.1002/eji.201142048 22213045

[34] Kollias G, Papadaki P, Apparailly F, Vervoordeldonk MJ, Holmdahl R, Baumans V, et al Animal models for arthritis: innovative tools for prevention and treatment. Ann Rheum Dis 2011 8;70(8):1357–1362. 10.1136/ard.2010.148551 21628308

[35] Salvarrey-Strati A, Watson L, Blanchet T, Lu N, Glasson SS. The influence of enrichment devices on development of osteoarthritis in a surgically induced murine model. ILAR J 2008 9 1;49(4):23–30. 18849588 10.1093/ilar.49.3.e23

[36] Fu Y, Foden JA, Khayter C, Maeder ML, Reyon D, Joung JK, et al High-frequency off-target mutagenesis induced by CRISPR-Cas nucleases in human cells. Nat Biotechnol 2013 9;31(9):822–826. 10.1038/nbt.2623 23792628 PMC3773023

[37] Tse HM, Thayer TC, Steele C, Cuda CM, Morel L, Piganelli JD, et al NADPH oxidase deficiency regulates Th lineage commitment and modulates autoimmunity. J Immunol 2010 11 1;185(9):5247–5258. 10.4049/jimmunol.1001472 20881184 PMC3190397

