# Supplementary material for: Direct Comparison of a Natural Loss-Of-Function Single Nucleotide Polymorphism with a Targeted Deletion in the Ncf1 Gene Reveals Different Phenotypes
Source: PLoS One. 2015 Nov 3;10(11):e0141974. doi: 10.1371/journal.pone.0141974 (PMC4631371; doi:10.1371/journal.pone.0141974)
Supplement: S1 Fig — (PDF) [file pone.0141974.s002.pdf]

Supporting Information

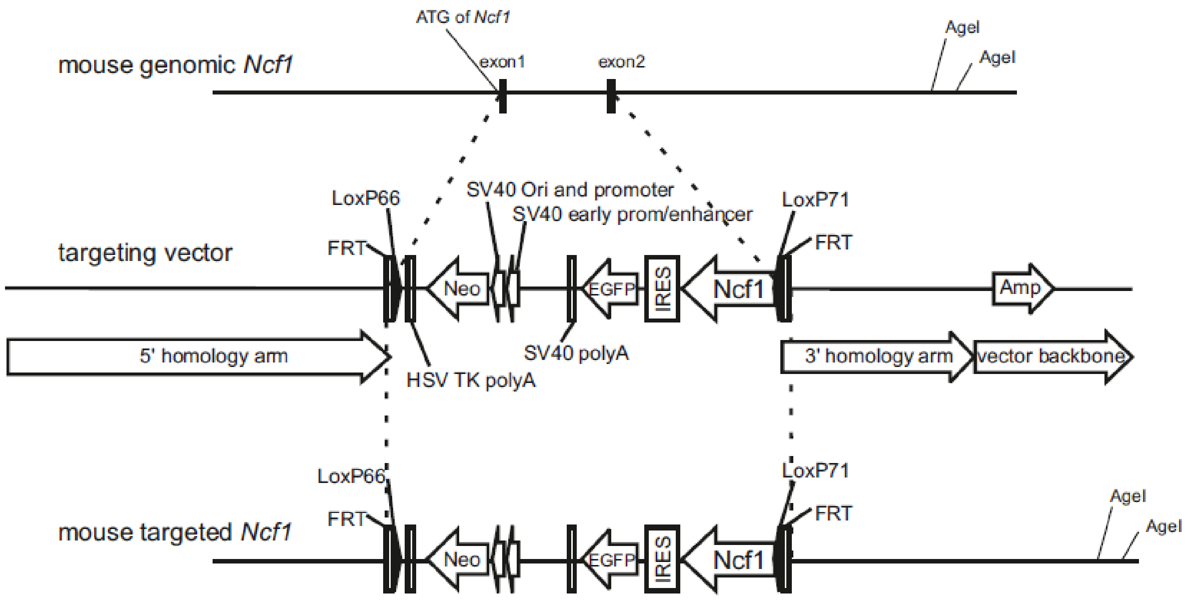

S1 Fig. The targeting strategy of the *Ncf1* (neutrophil cytosolic factor 1) gene. The targeting vector was incorporated into the genome by homologous recombination, thus deleting exons 1-2 in the *Ncf1* gene resulting in an *Ncf1* gene knockout. The vector contained the *Ncf1* cDNA followed by IRES and EGFP sequences as well as the Neomycin resistance gene (Neo) in reverse orientation in respect to the *Ncf1* promoter, thus keeping the cassette silent in the genome. The cassette was flanked with both LoxP and FRT sites to enable conditional genome modifications in case crossed with strains expressing Cre recombinase or FLP (flippase), respectively.

EGFP, enhanced green fluorescent protein cDNA; IRES, internal ribosome entry site; Amp, ampicillin resistance; SV40, Simian virus 40; HSV TK, herpes simplex virus thymidine kinase.
