# Supplementary material for: Direct Comparison of a Natural Loss-Of-Function Single Nucleotide Polymorphism with a Targeted Deletion in the Ncf1 Gene Reveals Different Phenotypes
Source: PLoS One. 2015 Nov 3;10(11):e0141974. doi: 10.1371/journal.pone.0141974 (PMC4631371; doi:10.1371/journal.pone.0141974)
Supplement: S2 Fig — (PDF) [file pone.0141974.s003.pdf]

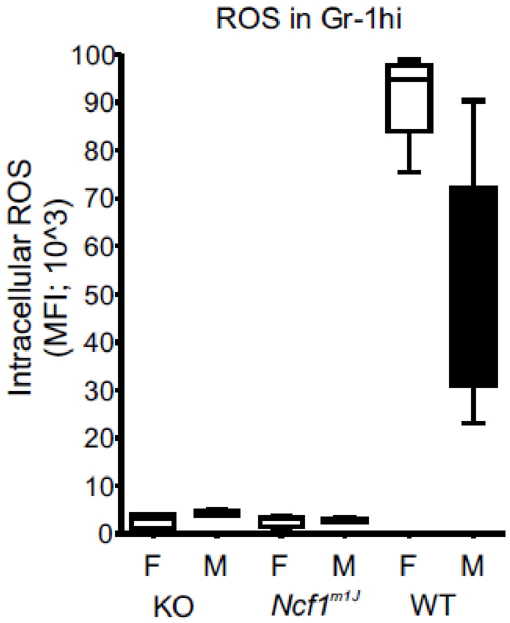

S2 Fig. ROS production by *Ncf1* deficient (KO and *Ncf1*^{m1J}) and the wild type mice during CIA.

Blood was collected 71 days after immunization and analyzed for intracellular ROS production. The data is presented as box-and-whisker blot showing the quartiles. Number of mice per genotype: n=4-6.
