# Supplementary material for: Direct Comparison of a Natural Loss-Of-Function Single Nucleotide Polymorphism with a Targeted Deletion in the Ncf1 Gene Reveals Different Phenotypes
Source: PLoS One. 2015 Nov 3;10(11):e0141974. doi: 10.1371/journal.pone.0141974 (PMC4631371; doi:10.1371/journal.pone.0141974)
Supplement: S3 Fig — (PDF) [file pone.0141974.s004.pdf]

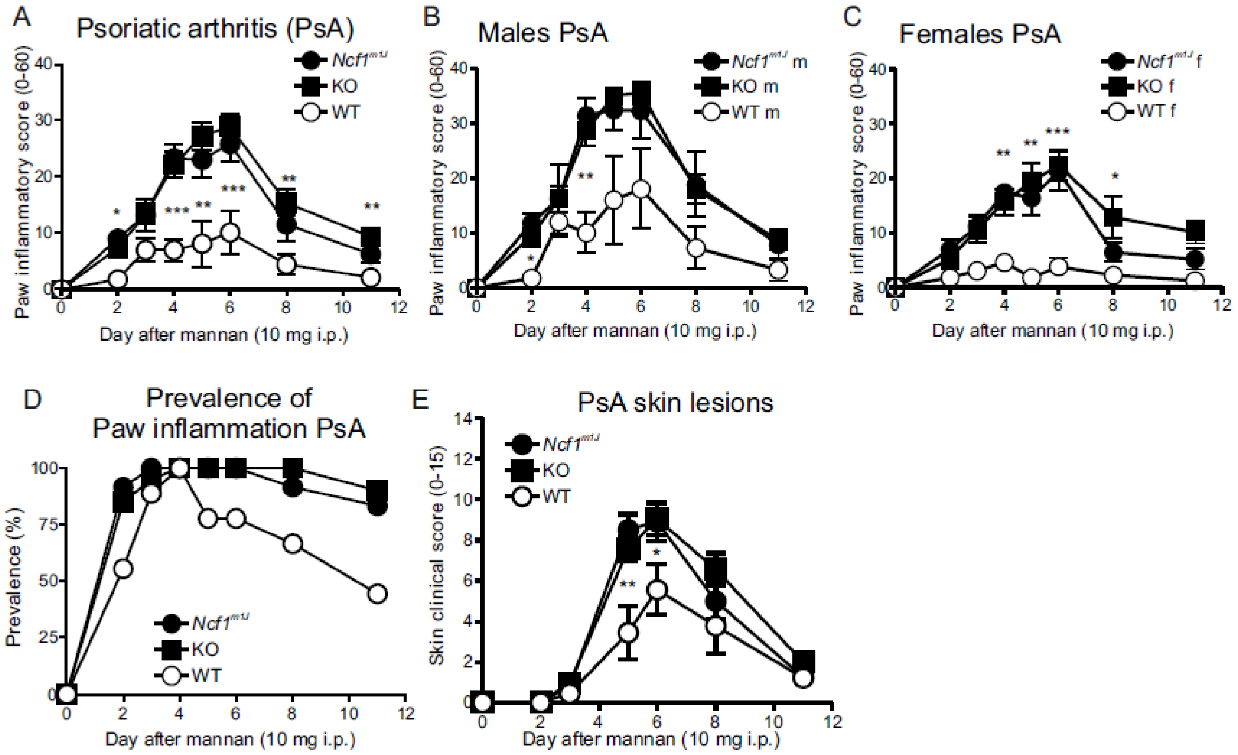

S3 Fig. ***Ncf1*** **deficient (KO and** $Ncf1^{m1J}$**) mice develop similar disease when exposed to psoriatic arthritis (PsA).** PsA was induced by mannan and the paw inflammation was evaluated by a macroscopic scoring system (see methods for details) and presented as both sexes combined (A), and splitted (B, C), males and females, respectively. The prevalence of paw inflammation (D) and the severity of the skin lesions (E) are shown. The wild type group comprises of both homozygously or heterozygously wild type animals. Significances were calculated between the *Ncf1* KO and wild type group using Mann-Whitney U-test and presented as *** $P<0.001$, ** $P<0.01$ and * $P<0.05$. n=9-20 in A and D-E, n=4-10 in B, n=5-10 in C.
