# Supplementary material for: Direct Comparison of a Natural Loss-Of-Function Single Nucleotide Polymorphism with a Targeted Deletion in the Ncf1 Gene Reveals Different Phenotypes
Source: PLoS One. 2015 Nov 3;10(11):e0141974. doi: 10.1371/journal.pone.0141974 (PMC4631371; doi:10.1371/journal.pone.0141974)
Supplement: S1 Table — (PDF) [file pone.0141974.s005.pdf]

S1 Table. CIA in B6N;B10.Q mice with *Ncf1* deficiency.

Group	n	Mean age (weeks) +/-SD	Median Age (weeks) (Min-Max)	Prevalence	Day of onset mean (+/-SEM) [a]
**Experiments I-II**					
$Ncf1^{m1J}$ all	34	12 +/- 3	12 (7-19)	27/34 (79 %)	38.9 (2.3)
KO/$Ncf1^{m1J}$ all	39	11 +/- 4	11 (7-19)	24/39 (62 %)	46.3 (2.4)
KO all	32	11 +/- 4	10 (7-19)	20/32 (63 %)	43.9 (2.7)
WT all	9	9 +/- 2	9 (7-12)	0/9 (0 %)	
$Ncf1^{m1J}$ m	21	12 +/- 3	11 (7-19)	16/21 (76 %)	39.6 (3.0)
KO/$Ncf1^{m1J}$ m	25	11 +/- 3	11 (7-19)	18/25 (72 %)	46.4 (2.9)
KO m	16	12 +/- 4	12 (7-19)	13/16 (81 %)	36.9 (2.4)
WT m	5	9 +/- 2	9 (7-12)	0/5 (0 %)	
$Ncf1^{m1J}$ f	13	12 +/- 3	12 (7-15)	11/13 (85 %)	37.7 (3.8)
KO/$Ncf1^{m1J}$ f	14	11 +/- 4	9 (7-19)	6/14 (43 %)	42.3 (5.0)
KO f	16	9 +/- 3	8 (7-15)	7/16 (44 %)	56.0 (1.5) **
WT f	4	10 +/- 2	11 (7-12)	0/4 (0 %)	
**Experiment I**					
$Ncf1^{m1J}$ all	11	9 +/- 2	8 (7-12)	10 /11 (91 %)	32.2 (3.9)
KO/$Ncf1^{m1J}$ all	11	9 +/- 2	8 (7-12)	6 /11 (55 %)	34.7 (4.5)
KO all	11	8 +/- 2	8 (7-12)	8/11 (73 %)	40.1 (4.0)
WT all	9	9 +/- 2	9 (7-12)	0/9 (0 %)	
$Ncf1^{m1J}$ m	6	9 +/- 2	8 (7-11)	5/6 (83 %)	32.8 (6.5)
KO/$Ncf1^{m1J}$ m	7	9 +/- 2	8 (7-12)	3/7 (43 %)	33.7 (4.8)
KO m	6	8 +/- 2	8 (7-12)	5/6 (83 %)	34.4 (1.7)
WT m	5	9 +/- 2	9 (7-12)	0/5 (0 %)	
$Ncf1^{m1J}$ f	5	9 +/- 2	8 (7-12)	5/5 (100 %)	31.6 (5.0)
KO/$Ncf1^{m1J}$ f	4	9 +/- 2	8 (7-12)	3/4 (75 %)	35.7 (8.7)
KO f	5	7 +/- 0.4	7 (7-8)	3/5 (60 %)	54.5 (3.5)
WT f	4	10 +/- 2	11 (7-12)	0/4 (0 %)	
**Experiment II**					
$Ncf1^{m1J}$ all	23	13 +/- 3	15 (8-19)	17/23 (74 %)	42.8 (2.5)
KO/$Ncf1^{m1J}$ all	28	12 +/- 4	11 (7-19)	17/28 (61 %)	49.1 (2.4)
KO all	20	12 +/- 4	12 (7-19)	11/20 (55 %)	42.1 (2.9)
WT all	0	-	-	-	
$Ncf1^{m1J}$ m	15	13 +/- 3	15 (8-19)	11/15 (73 %)	42.7 (2.9)
KO/$Ncf1^{m1J}$ m	18	12 +/- 3	11 (8-19)	14/18 (78 %)	49.1 (2.9)
KO m	9	14 +/- 4	13 (10-19)	7/9 (78 %)	36.9 (2.4)
WT m	0	-	-	-	
$Ncf1^{m1J}$ f	8	13 +/- 3	15 (8-15)	6/8 (75 %)	42.8 (5.1)
KO/$Ncf1^{m1J}$ f	10	12 +/- 5	11 (7-19)	4/10 (40 %)	49.0 (2.0)
KO f	11	10 +/- 3	11 (7-15)	4/11 (36 %)	57.5 (2.4)
WT f	0	-	-	-	

[a] the mean of the disease onset (the day post immunization). Mice that never developed any symptoms were excluded from the analysis. Statistical significances were calculated by One way ANOVA with Dunnett posttest. **P<0.01 when compared with $Ncf1^{m1J}$ females. m=males, f=females.
